# Human Stress and StO2: Database, Features, and Classification of Emotional and Physical Stress

**DOI:** 10.3390/e22090962

**Published:** 2020-08-31

**Authors:** Xinyu Liu, Yuhao Shan, Min Peng, Huanyu Chen, Tong Chen

**Affiliations:** 1 Chongqing Key Laboratory of Non-Linear Circuit and Intelligent Information Processing, Southwest University, Chongqing 400715, China; liuxinyu1223@email.swu.edu.cn (X.L.); fgyuhao@email.swu.edu.cn (Y.S.); peng2014m@email.swu.edu.cn (M.P.); chy199315@email.swu.edu.cn (H.C.); 2 School of Electronic and Information Engineering, Southwest University, Chongqing 400715, China; 3 Key Laboratory of Cognition and Personality (SWU), Ministry of Education, Chongqing 400715, China; 4 Institute of Psychology, China Academy of Sciences, Beijing 100101, China

**Keywords:** human stress database, hyperspectral imaging, tissue oxygen saturation, human stress classification, emotional stress, physical stress

## Abstract

Emotional and physical stress can cause various health problems. In this paper, we used tissue blood oxygen saturation (StO2), a newly proposed physiological signal, to classify the human stress. We firstly constructed a public StO2 database including 42 volunteers subjected to two types of stress. During the physical stress experiment, we observed that the facial StO2 right after the stress can be either increased or decreased comparing to the baseline. We investigated the StO2 feature combinations for the classification and found that the average StO2 values from left cheek, chin, and the middle of the eyebrow can provide the highest classification rate of 95.56%. Comparison with other stress classification method shows that StO2 based method can provide best classification performance with lowest feature dimension. These results suggest that facial StO2 can be used as a promising features to identify stress states, including emotional and physical stress.

## 1. Introduction

Stress, which refers to a state of tension when individuals are mentally or physically threatened, is a kind of imbalanced homeostasis state. Stress can be divided into psychological stress (also known as emotional stress) and physical stress according to different sources of stressors. Emotional stress comes from internal stimuli, such as the nervousness brought by stage speeches and important exams. Being under emotional stress for a long time is a chronic health challenge to the body. Serious emotional stress can cause some mental illnesses, such as depression. Physical stress comes from external stimuli, such as excessive exercise, working or driving long hours [1]. Short-term physical stress gives people a feeling of exhaustion and listlessness. Being under long-term physical stress will seriously affect our health. Epidemiological studies have pointed out that stress stimulus has a certain relationship with the increased incidence of hypertension, ulcers, accidents, cardiovascular events [2,3], myocardial infarction, diabetes, immunological problems and cancer [4,5,6,7].

Since both types of stress have an impact on health, the research of stress detection has attracted the attention of both engineers and psychologists. Depending on the source of signal, human stress detection can be based on external visual appearance as well as internal physiological signals. External appearance features can be extracted from facial expressions, gesture, voice, etc. Internal physiological features can be extracted from common physiological signals, such as ECG, EMG, EEG, breath, respiratory, etc. Although the stress detection based on external appearance is intuitive, it is also easily to be disguised, resulting in the failure of detection. Nevertheless, the internal physiological signals are involuntary and hard to manipulate, and usually correspond to true feelings. Therefore, it is more accurate to use physiological signals for stress detection.

In traditional studies, the stress detection based on physiological signals mostly uses contact methods [1,8,9,10,11]. In the process of this detection, participants need to wear sensors, which leads to inconvenience in operation and difficulty in promotion and application. In order to improve the convenience, stress detection based on imaging technology has been developing rapidly in recent years. Using some specific imaging technologies to extract physiological signals to detect stress has also achieved good results. Pavlidis et al. [12,13] used thermal imaging (TI) to measure blood flow under skin surface and perspiration of nose to detect stress. McDuff et al. [14] used broadband imaging to measure heart rate variability (HRV) to detect stress. We [15] used hyperspectral imaging (HSI) technology to extract tissue blood oxygen saturation (StO2) to detect psychological stress. Later on Hong et al. [16] used multispectral imaging (MSI) technology to measure the physical stress.

StO2, one of the latest physiological indicators, is associated closely with stress. When an individual feels stressed, the hypothalamus-pituitary-adrenal (HPA) secretes adrenaline, preparing the body for fight-or-flight. When in a state of stress, heart and lung activity increases, and nutrients such as glucose and oxygen are released in large quantities for muscle activity. At the same time, the blood of the whole body will also have corresponding changes. For example, blood pressure increases, blood thickens, and blood flow speeds up to 300~400% [15] for aroused brain, heart, and muscles. The response to stressors will bring about a series of changes in physiological indicators, including facial StO2, which is the basis of this study [15].

Most of the stress detection studies mentioned above focus on one type of stress recognition. However, physical stress and emotional stress share some similar characteristics, such as increased heart rate [15] and breathing rates, muscle tension [17], perspiration [12], which makes it somewhat difficult to distinguish between physical and emotional stress. Meanwhile, relief methods for the two types of stress are completely different [18]. Emotional stress requires proper psychological counseling, while physical stress requires adequate rest and relaxation. It is easy to confuse the two types of stress when the content of stimuli is unknown, or when the selection of physiological features is not appropriate. It is necessary to accurately distinguish between physical stress and emotional stress, and to take the problem-oriented strategy for different types of stress.

One of key factors for distinguishing these two types of stress would be to understand the characteristics of these two, i.e., to find out the distinguishing features among these two. We have investigated [19,20] one of traditional physiological signals, the respiratory signal, to find out some useful features for distinguishing these two types of stress. However, as regard to the latest physiological signal, the StO2, little work has been done to find out their distinguishing features. In a previous study [15], we found that when the participants were under psychological stress, the average StO2 values of their forehead were significantly higher than those of the baseline condition. In another pilot study [21], we found that the change trend of StO2 was different among different participants under physical stress. Some persons have higher StO2 right after the physical stress than that of baseline, while some have lower ones.

In this paper, we would like to examine in depth the distinguishing StO2 features of these two and differentiate the two types of stress only by using StO2 features. To achieve this, we firstly build an StO2 database including 42 participants’ facial StO2 data, under emotional stress, physical stress, and baseline states. This database is the first StO2 database including two types of stress and will be publicly available to the research community. We then carefully examine the facial regional StO2 when participants are in stress. The changes of StO2 due to stress vary from person to person and from face region to region. After that the best combination of regional StO2 were determined by using exhaustive search. By using the group of regional StO2, the emotional stress and physical stress are differentiated. The average accuracy of classifying the two types of stress reaches 95.56%.

To sum up, the main contributions of this paper have three aspects. Firstly, we provide the first public stress database of StO2 through non-contact HSI, which contains emotional stress and physical stress. Secondly, we found that the changes of facial StO2 vary in different face regions and different persons, and the type of stress also make difference. Thirdly, we tested all the combinations of the seven regional StO2 features to find the optimal StO2 feature combination, and classified the two types of stress by only using StO2 features.

The structure of the rest paper is arranged as follows: Section 2 shows the experimental design and the establishment of StO2 stress database. Section 3 explores different facial StO2 changes under different types of stress. Section 4 presents the detection of emotional stress and physical stress, including the detection results and the study of certain feature regions. Conclusion and discussion are given in Section 5.

## 2. StO2 Stress Database

### 2.1. Generating Facial StO2 by HSI

HSI is a technique that performs detailed segmentation on spectral dimensions and enables imaging in hundreds of adjacent narrow bands with a bandwidth of about 10 nm. What is obtained by HSI is a 3D image cube [22,23,24], which contains not only spatial (two-dimensional) but also spectral (one-dimensional) information. The traditional imaging technique is to image on three broad-band wavelength channels (R,G,B), and tends to reduce the color discrimination ability. However, HSI can be performed in hundreds of narrow wavebands, with a strong ability to distinguish colors, coupled with the texture information of the object, making it has a strong ability to distinguish materials [25]. It is based on this characteristic of HSI that we use it to sense and distinguish blood chromophores inside body tissues [26].

When air is inhaled into the lungs, oxygen diffuses in the lung to the blood and binds to deoxygenated hemoglobin to form oxyhemoglobin (HbO_2_) in an unstable and reversible way. When the oxygen detached to diffuse in the cell, the HbO_2_ complex becomes into deoxygenated hemoglobin (Hb). Hb returns to the heart through the veins and then enters the lungs to bind to the inhaled oxygen again. The body’s entire blood will carry out such circulation process. Each hemoglobin molecule can bind to up to four oxygen molecules. Hemoglobin oxygen saturation (SO2) is defined as the ratio of the amount of HbO_2_ to the total amount of hemoglobin:

(1) $$\mathrm{SO}2=\frac{HbO_{2}}{Hb+HbO_{2}}$$

The StO2 used in this paper is the SO2 of microcirculation in tissues, ranging from approximately 60% of venous SO2 to 98% of arterial SO2 [15,27,28]. When the individual performs strenuous exercise or experiences extreme emotions such as fear, the value of StO2 will deviate from the normal level. For example, the facial StO2 during exercise could be less than 60%. Sufficient work has been done to extract StO2 data by HSI based on Beer-Lambert Law [29,30,31]. The facial StO2 in this paper is deduced from HSI date by using Modified Beer Lambert Three-Chromosphere model [15,24].

### 2.2. Experimental Setup

The HSI system utilized in this study consists of a Specim VNIR spectrograph (SPECIM, SPECTRAL IMAGING LTD., Oulu, Finland) together with a PixelFly camera (PCO, PCO AG, Kelheim, Gemany) and a home-designed mirror scanning system. The slit of the spectrograph is 30 μm width giving a maximum spectral resolution of 2.8 nm. The limit of the spectral sensitivity of the PCO camera ranges from 400 nm to 1000 nm with a maximum quantum efficiency yield of 65% at 650 nm. The size of whole HSI system is about 40 cm × 20 cm × 12cm. The HSI system images at a fixed number of wavelengths, i.e., from 380 nm to 800 nm with a step of 2 nm (300 wavelengths). It needs 20 s for the system to record one image cube.

During the whole imaging process, halogen lights were used as the sole illumination source. The HSI system is about 1 m away from the participants. The HSI data were obtained when the participants were in still pose. During the imaging, the participants were required to rest their jaws on a jaw bracket just like the ones used in optometry to keep the face still. Twenty seconds are required for the system to record one image cube. Any slight movement of the participants will cause distortion of the StO2 image, however, this distortion will be adjusted by using a registration operation.

### 2.3. Experimental Procedures and Protocols

**Figure 1:**
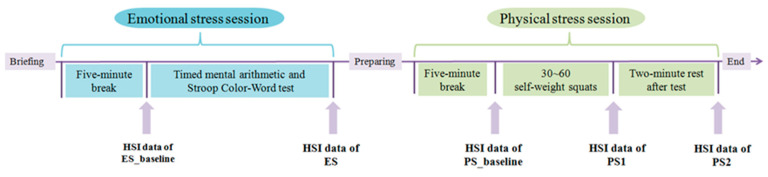
Experimental procedure.

Forty-two volunteers participated in the experiment. They were students aged from 18 to 25 at Southwest University, half of whom were male and half female. All participants were healthy, with no color recognition disorder and free from mental and physical illnesses. Prior to the experiments, everyone signed an informed consent. The experimental protocols were approved by the local Ethics Committee of Southwest University. All the experiments were conducted indoors with the room temperature being 26 degrees Celsius (air conditioning was applied), and the data were collected while the participant was sitting in a chair. The participant was given 10 min to adapt after entering the experimental environment. During the 10 min, the participant was introduced the procedure details of the experiment (see Figure 1). The experiment consisted of three main sessions: the baseline session, the emotional stress session and the physical stress session. A modified Trier Social Stress Test (TSST) was used to stimulate participants’ emotional stress. The TSST is a widely used social psychological stress test in the world [32]. Its psychological stress test includes public speaking and numerical calculation. Since there is no professional audience in our experiment, we used a Stroop Color-Word test [33] to replace the public speech. In this paper, timed mental arithmetic and Stroop Color-Word tests [33] were employed as emotional stressors, and each stimulus was randomly presented. Studies have shown that Stroop Color-Word test can effectively stimulate participants’ psychological stress [34,35]. The Stroop Color-Word test slide shows a color-meaning word (Red, blue, green or yellow) in colors that differ from the meaning of the word, such as the word “Green” in Blue. Participants were asked to randomly choose the meaning or color of the word. Before the test, the participant was told that if he/she made more correct choices than the average, he/she would get CNY20 as a reward. The answer time per slide was 5 s at the beginning, and gradually decreased to 3.5 s at the end of the test to increase the difficulty. HSI data of emotional stress was collected as ES. In the physical stress session, participants were asked to perform 30 to 60 self-weight squats as many as they could. After the squats, they returned to their seats immediately and we obtained the facial HSI data of physical stress as PS1. Physical stress data PS2 was taken after the participant had rested for two minutes after the squats. Before each session, there was a baseline session, during which participants were given a five-minute break to adjust their states back to normal. Then HSI data of baseline was collected. After all sessions were completed, participants were asked to evaluate the levels of emotional and physical stress on a scale of 1 to 10. A self-reported score greater than 6 was considered as valid data for subsequent analysis and identification. In our experiments, all participants reported stress levels greater than 6. HSI data were collected at the end of each experimental session. The baseline data corresponding to each session were collected to remove individual differences.

The emotional stress test was performed first and then the physical stress was performed (See Figure 1). The sequence of these two tests was not randomized. To eliminate the any possible effect of emotional stress test on the physical stress, there are five minutes for the participant to prepare the physical stress and another five minutes to break so as to restore himself/herself back to normal. That is to say from the end of the emotional stress test to the beginning of physical stress, there are ten minutes for the participant to rest. Another way to ensure the independence of these two tests or sessions is to take different baseline StO2 for the two tests. Right before the emotional stress and physical stress, the HSI data was recorded to generate emotional stress baseline StO2 (ES_baseline in Figure 1) and physical stress baseline (PS_baseline in Figure 1). Right after the stress test, the HSI data was recorded again to generate emotional stress StO2 (ES in Figure 1) and physical stress StO2 (PS1 and PS2 in Figure 1). The differences between baseline and stress StO2, ES-ES_baseline and PS-PS_baseline, were used to classify the two types of stress.

### 2.4. Processing of HSI Data

**Figure 2:**
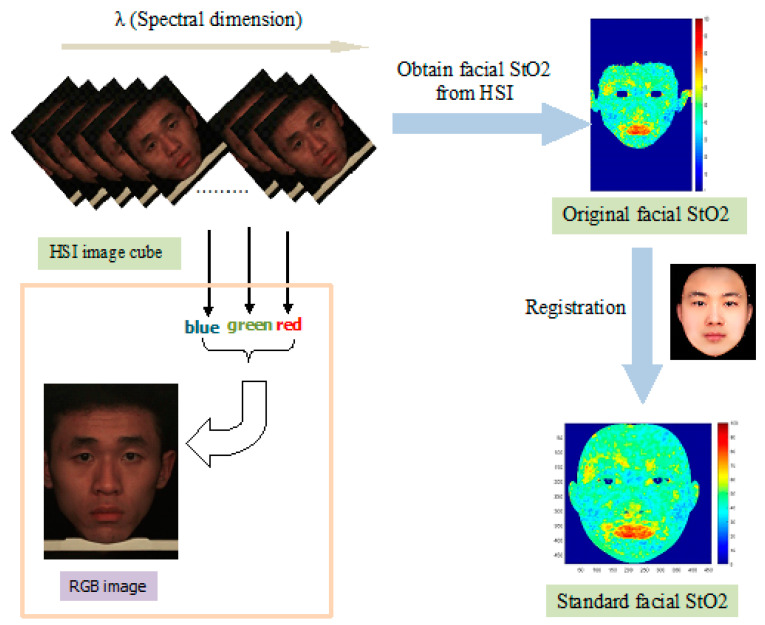
Processing flow of HSI data. The box in the lower left part indicates that R, G and B bands can be extracted from the HSI cube to form an RGB image.

The processing flow of HSI data is shown in Figure 2. The raw HSI date of faces were processed by using Modified Beer Lambert Three-Chromosphere model [15,24] to obtain facial StO2 data. Because each pixel of the StO2 image is affected by surrounding tissues, the final StO2 image was generated by averaging the raw StO2 image. For the convenience of subsequent use, we then registered the acquired facial StO2 image to the standard face through 65 key points of the face.

**Figure 3:**
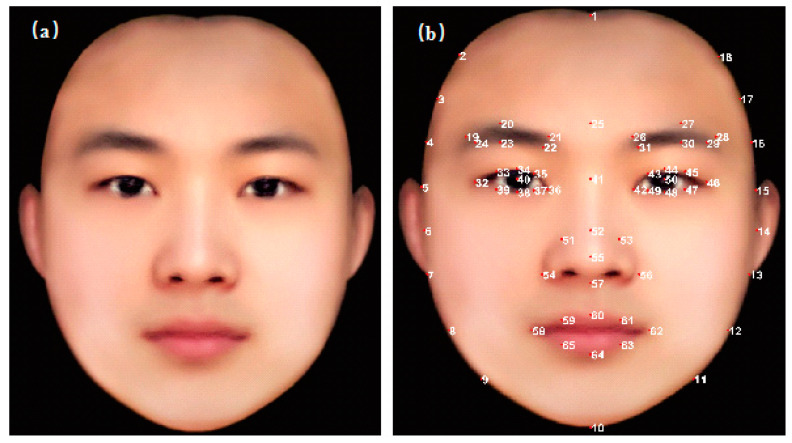
(**a**) The average standard face of 100 face images. (**b**) 65 facial key points for registration.

The standard face (see Figure 3a) is the average image of 100 students’ face images from Southwest University, which is generated by superimposing and averaging these 100 images through key points of the face. The StO2 registration process is as follows: Firstly, according to the facial 65 key points (see Figure 3b), we select the corresponding points on the original face image one by one to form a 65 × 2 matrix. Then, through geometric transformation, the original matrix is mapped to the same size matrix of 65 key points on the standard face, and a fourth-order polynomial transformation matrix is obtained. Finally, through this fourth-order polynomial transformation matrix, the original StO2 can be registered to the standard face.

By performing this processing, all the StO2 faces of different shape will become the same shape, so that the regional facial StO2 analysis can be conducted precisely (the size of the ROI of every StO2 face is the same). In our previous pilot study [21], we registered the face through 23 key points. In this paper, we used more key points for registration to achieve higher accuracy.

The ear region is not used in this research, therefore the key points do not include the points on the ear. The StO2 values in the final registered StO2 maps in ear regions are average StO2 values from the neighbor face regions and do not indicate real StO2 values of the ears.

### 2.5. Overview of StO2 Stress Database

The StO2 Stress database (Table 1) include stress data from 42 participants. Data for each participant include emotional stress data ES, physical stress data PS1, PS2, emotional stress baseline data ES_baseline, and physical stress baseline data PS_baseline. The data is facial StO2 image after registration, with the size of 513 × 911. Therefore, the StO2 Stress database include 210 (42 × 5) facial StO2 images. The baseline data can be used to remove individual differences. It is worth noting that due to the nature of the HSI imaging system mentioned earlier (see Section 2.2), some raw StO2 images may be distorted and stretched. However, after registration this distortion does not affect the subsequent analysis and use of data.

## 3. Facial StO2 of Stress

**Figure 4:**
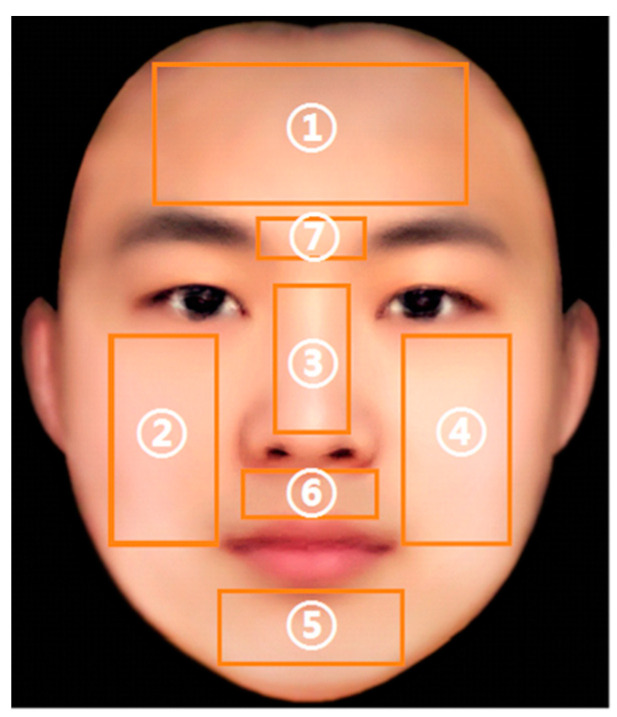
Illustration of seven selected ROIs.

In this research, seven regions of interest (ROIs) were selected to explore the changes of StO2 under different types of stress, i.e., forehead [15], left cheek, nose, right cheek, chin, the middle of the eyebrow (meixin) [16,21], and philtrum [16,21]. Within these ROIs, two [16,21] of them have been investigated under solo type of stress. The rest five will be examined for the first time in this paper. The seven regions selected cover most of the upper, middle and lower parts of the face, which may fully represent facial StO2 features. The selected ROIs and their numberings are shown in Figure 4. Among them, ROI1 represents forehead, ROI2 represents the right cheek, ROI3 represents the nose, ROI4 represents the left cheek, ROI5 represents the chin, ROI6 represents the philtrum and ROI7 represents the meixin. Because all the StO2 maps were registered in a standard face, the area of each ROI of each participant is the same. When the HSI camera took a face image, the left part of the image collected was actually the right part of the participant, and no image mirror flip was performed in the subsequent processing. So ROI2 represents the right cheek of the participant, and ROI4 represents the left cheek of the participant. The mean StO2 (M_StO2) value within each ROI calculated. In the study of facial StO2 changes under different stress states, we selected typical data of six participants for analysis. Of course the StO2 figures of all the 42 participants can be accessed in our published database. The data of the six participants selected are named from sub1 to sub6, and the corresponding ID of these participants in the database is 2, 15, 19, 10, 23 and 29, respectively.

### 3.1. Facial StO2 of Emotional Stress

**Figure 5:**
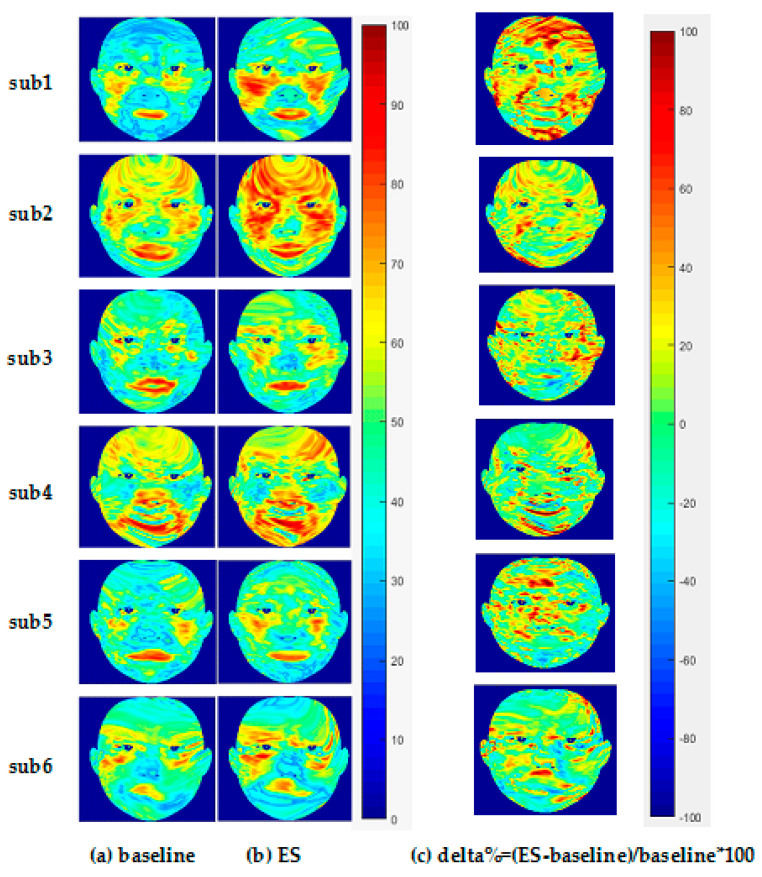
Facial StO2 maps of six participants under (**a**) baseline and (**b**) emotional stress. (**c**) delta% represents the incremental percentage of facial StO2 under emotional stress.

**Figure 6:**
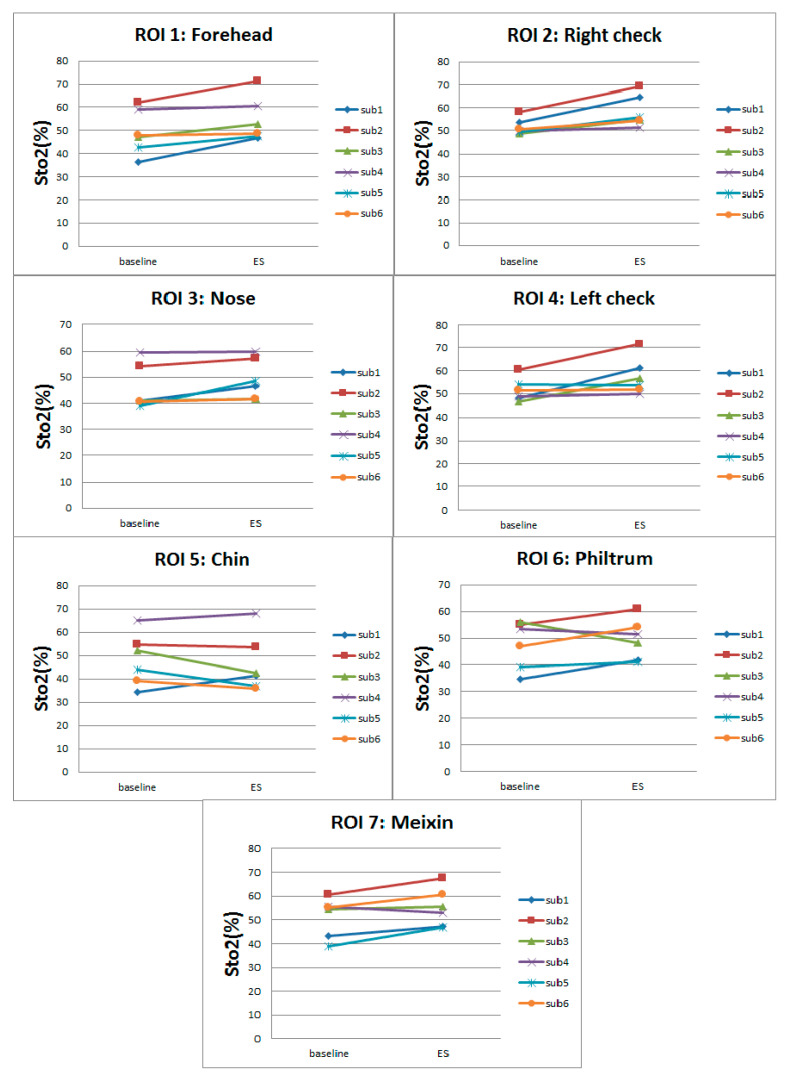
Average facial StO2 value of seven ROIs under baseline and emotional stress.

The left part of Figure 5 shows the facial StO2 of six participants under baseline state and ES state, and right part shows the delta% of ES StO2, with the warm color representing high StO2 value and high delta% (see color bar of Figure 5). Figure 6 shows the average StO2 values within ROIs under baseline state and emotional stress state. From Figure 5, it is seen that the ES facial StO2 are higher than the baseline facial StO2 visually (more warm color is presented). Quantitative analysis of change of StO2 due to ES from Figure 6 indicates that StO2 rises in the forehead region under emotional stress, which is consistent with our previous research [15]. Meanwhile, the left and right cheeks and the meixin region (except for sub 4) also rise significantly. However, compared to other regions, the increase of StO2 in the nose region was small or even unchanged (see Figure 6). StO2 in the chin region even decreased in three of these six participants (sub3, sub5, sub6).

**Figure 7:**
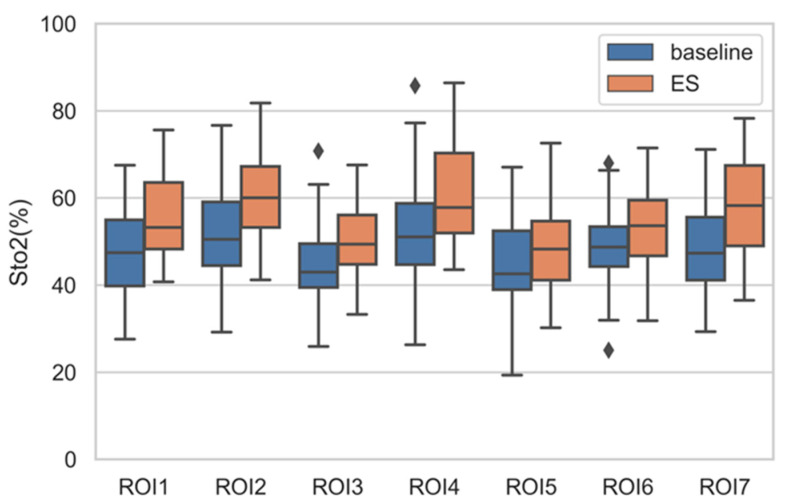
Boxplot of average StO2 of ROIs of all participants under ES and baseline.

**Figure 8:**
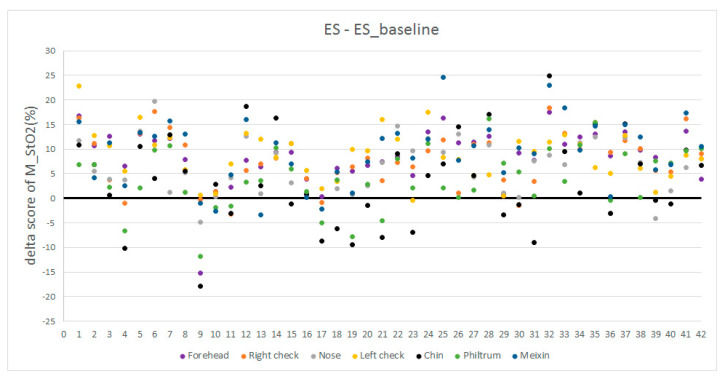
Delta score of M_StO2=ES-ES_baseline.

The average StO2 of every ROI of all participants can form a vector with 42 elements (42 participants). Performing statistical analysis on this vector (maximum, minimum, median, quartile), we can obtain a boxplot shown in Figure 7. It is observed that the ES StO2 are higher than baseline StO2 statistically. However, the StO2 within RIO3 (nose), ROI5 (chin), and ROI6 (Philtrum) have smaller change. At the same time, we calculate the delta score of M_StO2=ES-ES_baseline in seven ROIs of all participants under emotional stress, which are illustrated in Figure 8 with colors representing facial regions, numbers at horizontal axis representing ID of participants, and numbers in vertical axis representing delta score of M_StO2. It can be seen that most of the delta scores are larger than 0, which indicates that the ES StO2 trend is rising compared to the baseline. The delta scores that are smaller than 0 mostly belong to chin (black dots in Figure 8) and philtrum (green dots) areas, and the delta scores of nose areas (gray dots) are mostly near the 0, which indicate that the nose, chin and philtrum area will have smaller rising. These observation accords with the boxplot in Figure 7.

The increase of facial StO2 during ES could be due to the “fight-or-flight” state. When a person is under emotional stress, this “fight-or-flight” state could be triggered. The brain needs to function more quickly to prepare for the potential threat and the eye needs to see more sharply [36], therefore more oxygenated blood could flow into head through the neck artery. This oxygenated blood also floods the face and makes most of the facial areas experience higher StO2. The chin, philtrum, and nose areas are relatively small areas compared to the other facial areas. Most of lower StO2 during ES are observed in these three areas. We do not have a good physiological explanation for this observation. The small cases of lower StO2 in this area might be due to individual differences in the blood flow distribution mechanism. During the ES, the blood vessels of these areas are contracted and the blood flow to these three areas is restricted to supply more oxygenated blood to the more functional areas such as the eyes and brain. This phenomenon is similar to the “cold nose during stress”. When some persons are stressed, the blood flow to the nose can be reduced and thus they feel a cold nose.

### 3.2. Facial StO2 of Physical Stress

In our pilot experiment [21], in which 20 participants took physical stress tests, we found that some had lower overall PS1 data than baseline, while others had higher PS1 data. After a two-minute rest, the PS2 gradually rose towards baseline. In this paper, we increased the number of participants to 42, and still observed this phenomenon.

**Figure 9:**
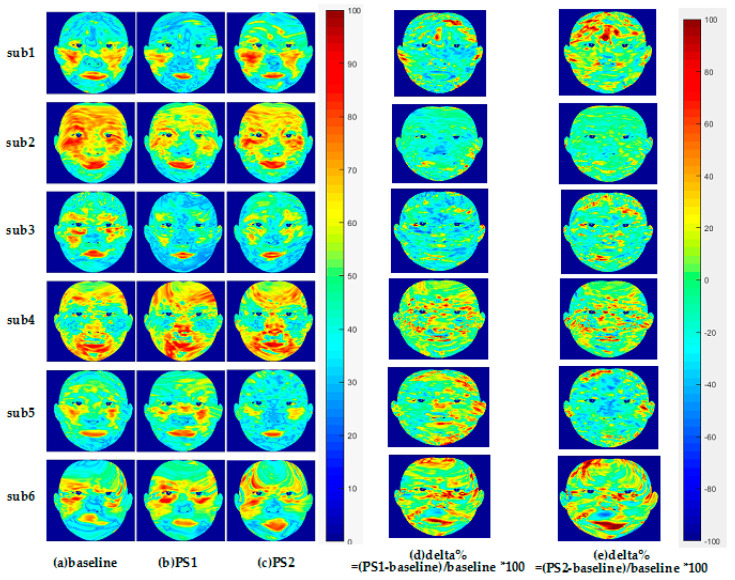
(**a**–**c**) Facial StO2 maps of six participants under physical stress. PS1 StO2 of the first three participants are lower than baseline StO2. (**d**,**e**) delta% represents the incremental percentage of facial StO2 under physical stress.

**Figure 10:**
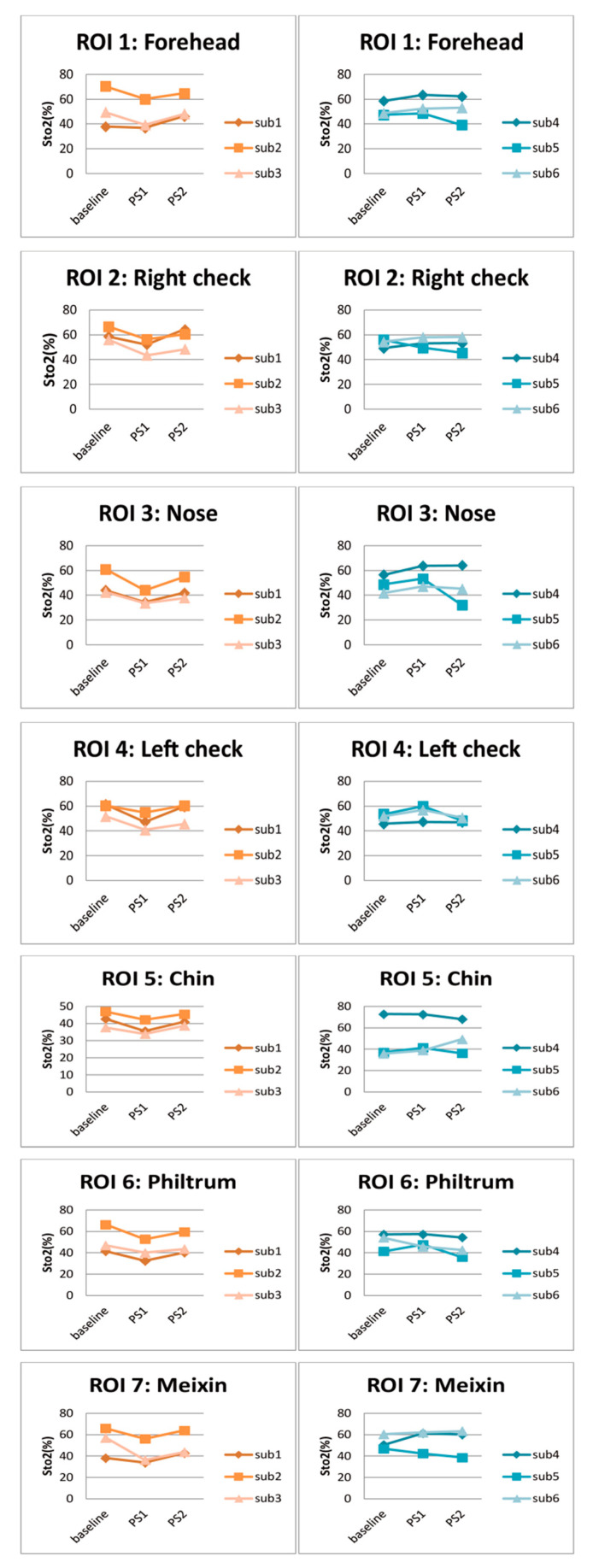
Average facial StO2 value of seven ROIs under baseline and physical stress. The left column represents PS1 StO2 lower than baseline StO2.

The left part of Figure 9 shows the facial StO2 of the six participants under baseline, PS1, and PS2 states, and right part shows the delta% of PS1 StO2 and PS2 StO2, respectively. Figure 10 shows the average StO2 values within ROIs under different states. It is seen from Figure 9 that three participants, i.e., sub1, sub2, sub3, had lower PS1 StO2 than the baseline, while for sub4, sub5, and sub6, their PS1 StO2 was higher than the baseline. These conclusions are reflected in the delta% (Figure 9d) that the most delta% colors of PS1 StO2 of sub1, sub2, sub3 are cold colors, while the most delta% colors of sub4, sub5, and sub6 are warm colors. Quantitative analysis from Figure 10 confirms the observation of Figure 9. All the PS1 StO2 values in the seven ROIs are lower than baseline in the three participants (left column of Figure 10). For the other three participants, forehead, nose, cheek (except for right cheek of sub5) all experienced little higher PS1 StO2.

**Figure 11:**
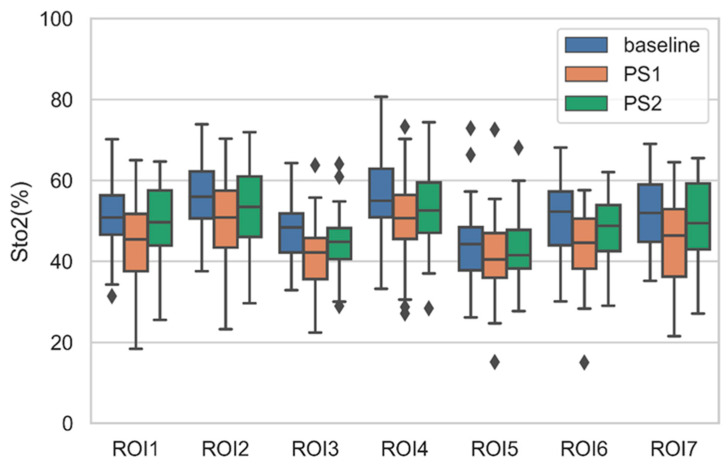
Boxplot of average StO2 of ROIs of all participants under PS and baseline.

By analyzing all 42 subjects, we found that more than 60% of the participants had lower PS1 StO2 than the baseline StO2. After a two-minute rest, the PS2 increases from PS1, which means PS2 changes towards the baseline value. On the other hand, other participants showed an overall increased PS1 compared to the baseline. The PS2 of these participants decreased compared to PS1, which means the PS2 also changes towards baseline. The boxplots of average StO2 of ROIs of all participants under PS and baseline are shown in Figure 11. The average PS1 StO2 is lower than average baseline StO2 in all ROIs. This is because more than 60% participants have lower PS1 StO2. The PS2 StO2 are all higher than PS1 and recover towards baseline.

PS1 StO2 is the StO2 collected when physical stress just finished, while PS2 StO2 is the StO2 collected when physical stress gradually dropped but had not completely disappeared. The different change trends of PS1 StO2 may thus be understandable. A familiar scene similar to this could be that different people may have different facial color changes right after a thousand-meter race. Some will turn pale and others will turn red. This difference may be due to the diversity of blood regulation mechanisms of individuals. When some persons exercise vigorously, the hearts beat faster and the breathing become faster. At this time, the cells of the body need a lot of oxygen support, which makes the blood vessels dilate and faces become red. However, for some other individuals, the ability of blood to carry and transport oxygen might be limited. When these people do exercise, most of the blood is supplied to the muscles, which causes the blood to the face to be reduced, resulting in the appearance of facial paleness.

**Figure 12:**
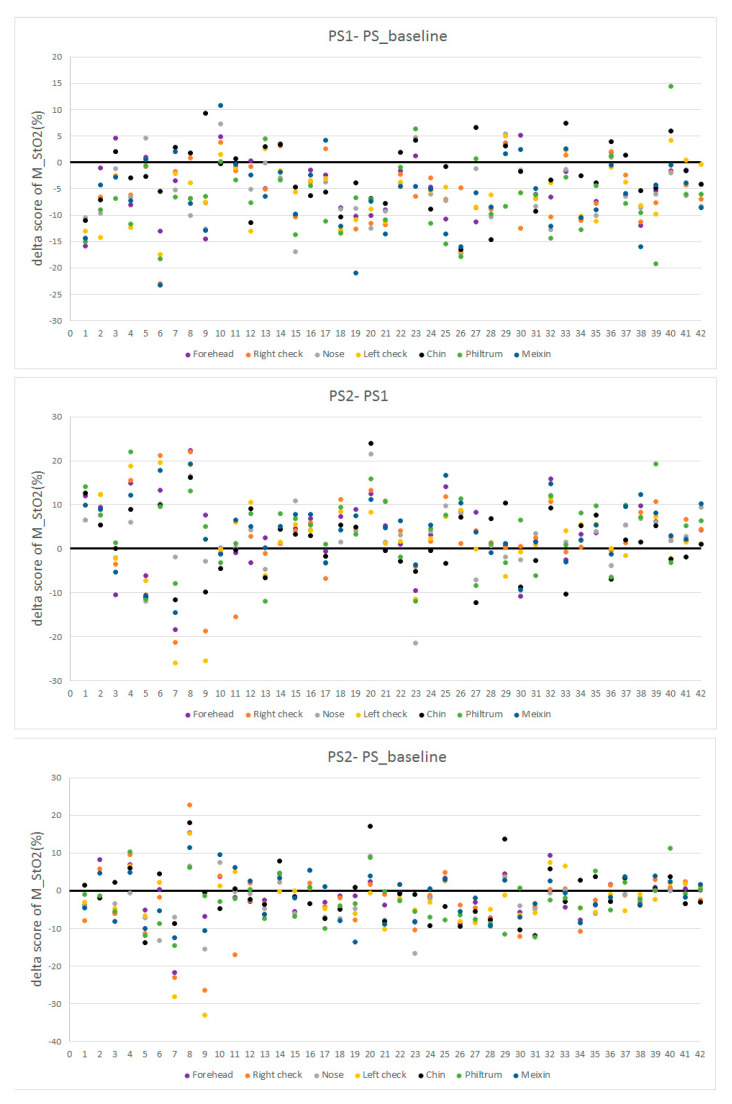
Delta score of M_StO2 = PS1−PS_baseline, M_StO2 = PS2−PS1 and, M_StO2 = PS2−PS_baseline.

We also calculated the delta scores of M_StO2 = PS1−PS_baseline, M_StO2 = PS2−PS1, and M_StO2 = PS2−PS_baseline in seven ROIs of all participants, which are illustrated in Figure 12 with colors representing facial regions, numbers at horizontal axis representing ID of participants, and numbers in vertical axis representing M_StO2. It is seen in Figure 12 that most data of M_StO2 = PS1-PS_baseline are smaller than 0, which confirms that the PS1 is lower than PS_baseline in most of the cases. For PS2-PS1, more than half of the values are larger than 0. The participants with more dots over 0 line in the PS1-PS_baseline (upper part of Figure 12) will have more dots below 0 line (middle part of Figure 12). For example, the participants 10, 29, and 33 have more dots over black line (0 values) in the PS1-PS_baseline plot, they also have more dots below the black line in the PS2-PS1 plot. This indicates that for those participants having higher PS1 than baseline, they will have lower PS2 than PS1, and their PS2 StO2 is changing towards baseline. This is also true for those participants with lower PS1 than PS_baseline, their PS2 are also changing towards baseline. By observing the PS2-PS_baseline in the lower part of Figure 12, we can see that more than 60% of the dots deviate more than 5% from the 0 value, which indicates that most participants still do not recover to baseline after 2 min rest. But comparing to the PS1-PS_baseline, the dots in PS2-PS_baseline are closers to the 0 value. There is no particular StO2 changing pattern, like that in the ES test, observed for specific facial areas in the PS test.

## 4. Stress Detection

### 4.1. Feature Extraction

Based on the analysis of ROI StO2, it is obvious that the value of ROI StO2 could be an indicator of stress. However, it is hard to determine which ROI or which combination of ROIs could be used to classify the two types of stress only by using statistic methods. Since there are seven selected ROIs, the number of the combination of ROIs is 7 + 21 + 35 + 35 + 21 + 7 + 1 = 127 (which is not large), we used an exhaustive search to find the best combination of ROIs that can give the highest classification rate.

For all participants, we took ES as the emotional stress data and PS1 as the physical stress data. The M_StO2 of ROIs were calculated as the features. StO2 may vary individually, it is influenced by adipose tissue thickness and gender [37]. Baseline information for each participant was also taken into account due to the individual differences among participants. The final features for the classification were M_StO2 of ES or PS1 minus M_StO2 of its corresponding baseline. Because each ROI produces one features, there are a total of 127 feature combinations (same as the number of ROI combinations). Each of 127 feature combinations was tried as input to a SVM classifier for the classification. The radial basis function (RBF) [38] was selected in SVM.

### 4.2. Feature Selection

Five-fold random cross-validation for 50 times was used in the classification task. The classification in this research is a two-class classification (either ES or PS). The database is well balanced, i.e., the number of ES samples is the same of the number of PS samples. Therefore, the classification rate is used as the object function, which is defined as the ratio of the number of predicted class to the number of actual class.

**Figure 13:**
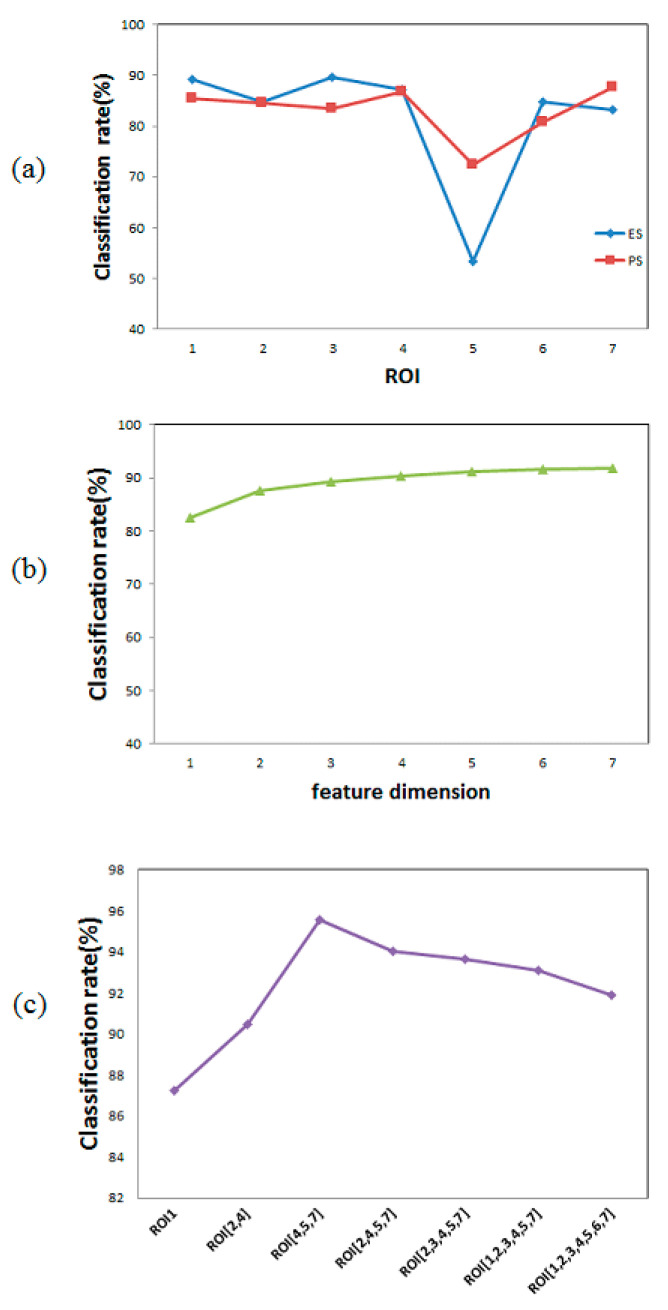
ROI combinations and classification rate. (**a**)The classification rate of every single ROI. (**b**)The average classification rate of combinations of ROIs in seven feature dimensions. (**c**) The maximum classification rate of combinations of ROIs in seven feature dimensions.

When the feature dimension is one (only one ROI), we can observe the effect of each ROI on the classification task. The average classification rate of every ROI is shown in Figure 13a. It is seen that the chin region (ROI5) had a poor effect on the discrimination of both stresses. The chin area only achieved an average recognition rate of 62.89% while the rest ROIs achieved an average recognition rate of more than 80%.

Figure 13b shows the average classification rate under different dimension size of features. When the feature dimension is 6, there are seven combinations of features, i.e., ROI{1,2,3,4,5,6} ROI{1,2,3,4,5,7}, ROI{1,2,3,4,6,7}, ROI{1,2,3,5,6,7}, ROI{1,2,4,5,6,7}, ROI{1,3,4,5,6,7}, and ROI{2,3,4,5,6,7}. The classification rate at feature dimension 6 in the Figure 13b is the average classification rate of these seven cases, which is 91.61%. It is observed from Figure 13b that as the dimension of features increases, the average of classification rate increases.

Figure 13c shows the best classification rate and combination of features within each feature dimension size. It is seen that the best classification rate at dimension size 6 is achieved by the combination ROI{1,2,3,4,5,7}, which is 93.09%. The highest classification rate (95.56%) of all 127 features combinations is achieved by combination ROI{4,5,7}.

Therefore, the selected ROIs/features are left cheek (ROI4), chin (ROI5) and meixin (ROI7). These three ROIs correspond to the middle, lower and upper facial areas of the whole face, so it is understandable that this combination can achieve a high recognition rate.

### 4.3. Cheek ROIs Comparison

The three selected ROIs correspond to the middle, lower and upper facial areas of the face. The middle part is the left cheek (ROI4). Because the blood vessels on both cheeks are symmetrical [39], it is natural to ask whether right cheek (ROI2) will have the same effect if it replaces the left cheek. The classification rates achieved by ROI4 and ROI2 are compared in Table 2. ROI4 can give 2% higher classification rate than ROI2 in both ES and PS situation. This finding might be related to the theory of division of labor between right and left brains [40,41,42,43].

Sperry’s split brain experiment in the late 1950s found that for the vast majority of right-handed people and the majority of left-handed people, the left hemisphere was associated with verbal, reasoning, rational and analytical thinking, while the right hemisphere was associated with perceptual, spatial subject perception and intuitive thinking. Therefore, the left brain could be called “conscious brain”, “academic brain” and “language brain”, while the right brain could be called “subconscious brain”, “creative brain”, “musical brain” and “artistic brain”. In our experimental process, stimulus sources of ES include timed mental calculation and Stroop Color-Word tests, both of which require rational calculation and analysis of participants. According to Sperry’s theory of the right and left brain, during the experiment of ES, the left brain activity increased, and the StO2 in the left cheek connected with the left brain nerve increased, resulting in the left cheek (ROI4) features showing considerable superiority to the identification of ES. In order to explore whether the StO2 in the left cheek (ROI4) was significantly higher than that in the right cheek (ROI2) under the ES condition, we performed a *t*-test.

However, the *t*-test results between StO2 of ROI2 and ROI4 under various affective states in Table 3 show that there is no statistical difference between these two areas. The values that we used to conduct the *t*-test are the features that we used to detect stress. The *h* in Table 3 is the result of test, where *h* = 0 means there is no statistical difference between two datasets and *h* = 1 means there is a difference. The *p* is possibility. The smaller the *p* value, the more the difference. In general, when *p* is less than 0.05, we believe that there is a statistical difference between the two datasets. The *ci* is confidence interval and the *t* is the *t*-test value. The results of the *t*-test directly indicate that the difference of StO2 changes in the left and right cheeks could not reach statistical significance under neither emotional stress nor physical stress. The observation in this research might also be because that the dataset is not large enough, the division theory does not apply in our study.

We have also examined the combination of ROI{2,5,7} and found this combination can only give a classification rate of 88.86%, much lower than what ROI{4,5,7} can achieve (95.56%). This observation from purely data analysis confirm that the left cheek is more suitable for stress detection in terms of achieving higher accuracy. The superiority of ROI {4,5,7} may came from the combination of ROI4 and the remaining two ROIs.

### 4.4. Classification of PS and ES

We used optimal ROI feature combination as input to some classical machine learning algorithms to classify the ES and PS, and the classification results were shown in Table 4. Except for the decision tree, all the classification rate achieved were more than 90%. In other words, the feature combination of the left cheek (ROI4), chin (ROI5), and meixin (ROI7) is robust for stress detection. All of the above results also indicate that stress can be identified and that the non-contact method of extracting StO2 features through HSI can effectively separate emotional stress from physical stress.

### 4.5. Comparison with Other Methods

Since there was no public HSI stress database before, there are no studies on using HSI to classify stress. Therefore, we cannot directly compare our results with those of others. However, we can compare achieved accuracy with that of the other methods, which have the similar goal with our task. The comparison is divided into three parts: the first part is the comparison with emotional stress detection, as shown in Table 5; the second part is the comparison with physical stress detection, as shown in Table 6; the third part is the comparison with the classification of emotional stress and physical stress, as shown in Table 7.

In related researches of emotional stress detection, some researchers [44,45,46] used physiological signals collected by the contact method to perform stress detection. Non-contact stress detection [14,15,20,47,48] had also achieved good results with the development of imaging technology. As shown in Table 5, among all methods, whether contact or non-contact, our proposed method achieved the highest accuracy (96.82%). Moreover, the number of features in our method is very small (only three features).

Compared with emotional stress detection, there are relatively few physical stress detection studies. Early physical stress detection mainly focused on drivers’ physical fatigue while driving [1,11]. In recent years, most physical stress detection research uses non-contact imaging-based methods [16,20,21,47]. The comparison of PS detection is given in Table 6. Among all the methods, our proposed method achieved the highest accuracy (94.3%) and the feature dimensions of our method are only three.

Very few studies have been performed to distinguish between emotional and physical stress. There are two papers that discriminate between emotional stress and physical stress. Both papers used a contactless measurement, which was similar to the task we did. Hong et al. [47] distinguished the two types of stress based on thermal signals. Our previous work [20] used respiratory signals based on a Kinect depth perception camera for the classification. The average accuracy of stress classification is shown in Table 7. The classification rate achieved in this paper is higher than that in [47], but lower than that in [20]. However, the dimension size used in this research is much smaller than that used in [20], but the same as that in [47].

## 5. Discussion and Conclusions 

In this study, we proposed to classify stress by using facial StO2 extracted from HSI. The way used to acquire HSI signals is a contact free method. Facial StO2 has been proved to be an excellent physiological indicator to distinguish stress. When using StO2 features to detect stress, only a few features can achieve a high accuracy.

We organized the data collected during the experiment into a public StO2 stress database, which contains two common stress categories, emotional stress and physical stress. Most studies so far have focused only on one type of stress detection. Our open stress database will provide researchers with a data platform to study and detect both types of stress.

By visualizing StO2 changes in seven major facial regions under two stress states, we observed different trends of StO2 change in these seven regions and the changes were also different among different persons. This indicates that the StO2 feature can effectively distinguish between the two types of stress. Moreover, by comparing the facial StO2 immediately after physical stress and two minutes later, we found that the change trend of StO2 among different participants is different. Some people had higher StO2 levels than baseline after physical stress, while others had lower ones. This is consistent with the phenomenon in one of our pilot studies [21]. Through data statistics, we found that PS1 StO2 below the baseline StO2 accounted for more than 60% of the total cases. However, this result is based on the fact that the participants are all young university students. This situation might change if more participants of different ages or jobs were added. The effect of age or exercise capacity on the change trend of PS StO2 is still unknown at this stage.

We tried all the feature combinations of the seven selected ROIs to classify stress and found the feature combination that can achieve the highest recognition rate of 95.56%, i.e., the left cheek, chin, and meixin. At the same time, it can be observed that under this optimal combination of features, if the left cheek feature was replaced by the right cheek feature, the classification rate will be greatly reduced. To understand this phenomenon, we conducted a series data analyses. It was found that there was no significant difference in StO2 between the left and right cheeks, and the accuracy difference came from the combination of features.

The StO2 can be influenced by adipose tissue and thus differs between males and females [37]. However, the effect of adipose tissue on StO2 has not been considered in this research. The input wavelengths of this study is from 518 nm to 580 nm [15]. In this wavelength range, the main chromophores are Hb, HbO2, and melanin, which have been considered in our StO2 producing model [15]. Pure mammalian fat starts to absorb light from around 700 nm and reaches absorption peaks at around 920 nm [49]. Though considering the effect of adipose tissue will provide more accuracy in the research, we have not taken this effect into account due to the wavelength used in this research being below 700 nm. The exact reading of StO2 in this research might therefore deviate slightly from the real reading. However, the input features to the classification model are the PS1-PS_basline, and ES-ES_baseline, which are the difference between stress StO2 and baseline StO2. That is to say, we depend on the change of StO2 not the exact reading of StO2 to classify the two types of stress. This operation could avoid the effect of the small deviation of StO2 readings, because since the deviation due to the adipose tissue for the same person is fixed, the difference between stress state and baseline states can eliminate the deviation.

The StO2 was produced by using HSI in this research. The HSI [22] is also a spectroscopy technique, and a complementary imaging option of the point/line spectroscopy. It uses reflectance or transmittance at every wavelength of materials to analyze the contents of the materials. This characteristic is the same as that of NIR spectroscopy. However, the HSI normally takes a wider range of light wavelengths as the input, such as visible to near-infrared (from roughly 380 nm to 2500 nm), while NIR spectroscopy uses input wavelengths from roughly 700 nm to 2500 nm. Another characteristics of HSI is that it can generate a 3D image cube, where every pixel in a 2D image has also spectral values for all the wavelengths.

The physical stress test in the research requires the participant to do as many squats as possible within a certain period of time to trigger physical stress, and the participant will report their stress level after the test. For the data of 42 participants, the self-assessment stress levels are all greater than 6 points (rating 1 to 10 points), which means that 30 to 60 squats can indeed trigger physical stress for these participants. The maximal exercise is not achieved in this study, the physical stress induced in the research is not the stress induced by maximal exercise. In that case, the self-assessment levels could be as high as 10.

The features used in the research are relatively simple and the classification task is two-class task (emotional stress or physical stress), we therefore choose SVM as the classifier because SVM is one of the simplest but classical classifiers suitable for two-class classification task. We also used other classical and commonly used machine learning algorithms, such as KNN and decision tree (see Table 4), for the classification. Compared to the SVM, they cannot achieve as high a classification accuracy as SVM. There will be of course other methods that can improve the classification accuracy. Researchers are encouraged to develop their own more sophisticated algorithms to reach higher classification rates on the published database.

To achieve high classification accuracy of stress detection, there are two factors. One is the algorithm used, the other is the input data. In this paper, what we would like to emphasize is that the input data (StO2) is a good signal for stress classification. To achieve this purpose, we have used a very simply algorithm to classify the two types of stress, i.e., calculating the average StO2 on ROIs as features, using SVM as the classifier. These algorithms are the simplest (or as simple as the others) in the Table 5 and Table 6. However, the algorithm can achieve the best classification accuracy. The results support our notion that facial StO2 can be used as a promising feature to identify stress states, including emotional and physical stress.

Since the importance of stress to human health cannot be ignored, it is essential to accurately detect stress and propose appropriate stress relief methods accordingly. In this paper, the three facial area features extracted by HSI can achieve an accuracy of more than 95% to distinguish between emotional stress and physical stress. This indicates that the facial StO2 feature is a valid physiological indicator of differentiating stress. The proposed method could be used to monitoring and discriminating human stress and suggest a proper treatment method according to the type of stress detected. It will also interesting to investigate in the future whether StO2 could be a potential feature for detecting basic emotions.

## Figures and Tables

**Table 1:** Overview of StO2 stress database.

Total Number of Participants	Stress Categories	Spatial Resolution of Each Image	Total Number of StO2 Images
42	Baseline of ES ES Baseline of PS PS1 PS2	513∗911	210

**Table 2:** Comparison of accuracy between ROI2, ROI4 and feature combination with ROI5 and ROI7.

Single Feature or Combination of Features	ES (%)	PS (%)	Average (%)
ROI2	84.71	84.61	85.66
ROI4	87.26	86.78	87.02
ROI{2,5,7}	85.78	91.94	88.86
ROI{4,5,7}	96.82	94.3	95.56

**Table 3:** T-test results of ROI2 and ROI4 features.

Features for *t*-Test	*h*	*p*	*ci*	*t*
All_ROI2,All_ROI4	0	0.3776	−1.6550,0.6340	−0.8872
ES_ROI2,ES_ROI4	0	0.4111	−2.4781,1.0340	−0.8304
PS_ROI2,PS_ROI4	0	0.6973	−1.8402,1.2423	−0.3917

**Table 4:** Recognition rate of other algorithms with the best ROIs’ features as the input.

Algorithms	Accuracy
Linear Discriminant	90.5%
Logistic Regression	91.98%
KNN	92.28%
Decision Tree	86.9%
Ensemble learning	92%

**Table 5:** Comparison of emotional stress detection.

Methods	Measurements	Amount of Features	Classifier	Accuracy
[46]	Wearable EEG devices	80	Gaussian SVM	80.32%
[14]	Five band DC for heart rate, breath rate, HRV	3	Linear SVM	85%
[15]	HSI for StO2 signal of forehead	1	Binary classifier	88.1%
[44]	Galvanic skin response	16	ANOVA test	89%
[45]	ECG, HRV	20	KNN, SVM	92.75%
[47]	Thermal signals	3	EM-CCA, BP	93.3%
[48]	Non-contact Bioradar for respiratory signals	3	3 layer perceptron	94.44%
[20]	Kinect for respiratory signals	56	SVM	94.76% ^1^
Ours	HSI for facial StO2 signals	3	SVM	96.82%

^1^ Results of classification for emotional stress and the other two states (physical stress and relaxation).

**Table 6:** Comparison of physical stress detection.

Methods	Measurements	Amount of Features	Classifier	Accuracy
[21]	HSI for facial StO2 signals	5	SVM	82.11%
[47]	Thermal signals	3	Back Propagation	93.3%
[16]	MSI for StO2 signal	2	MCDS, LSTM	93.3% ^1^
[20]	Kinect for respiratory signals	56	SVM	92.17% ^2^
Ours	HSI for facial StO2 signals	3	SVM	94.3%

^1^ The result is the average accuracy of PS for three different loads. ^2^ Results of classification of physical stress and the other two states (emotional stress and relaxation).

**Table 7:** Comparison of emotional and physical stress classification.

Methods	Measurements	Amount of Features	Classifier	Accuracy
[47]	Thermal signals	3	EM-CCA, BP	93.3%
[20]	Kinect for respiratory signals	56	SVM	97.93% ^1^
Ours	HSI for facial StO2 signals	3	SVM	95.56%

^1^ Results of classification of emotional stress and physical stress.
